# Telepharmacy Services: Present Status and Future Perspectives: A Review

**DOI:** 10.3390/medicina55070327

**Published:** 2019-07-01

**Authors:** Simone Baldoni, Francesco Amenta, Giovanna Ricci

**Affiliations:** 1 Telemedicine and Telepharmacy Center, School of Pharmaceutical Sciences and Health Products, University of Camerino, 62032 Camerino, Italy; 2 Section of Forensic Medicine, School of Law, University of Camerino, 62032 Camerino, Italy

**Keywords:** telepharmacy, community pharmacy, clinical pharmacy, telepharmacy of care, internet pharmacy, pharmaceutical care

## Abstract

*Background and Objectives*: The term “telepharmacy” indicates a form of pharmaceutical care in which pharmacists and patients are not in the same place and can interact using information and communication technology (ICT) facilities. Telepharmacy has been adopted to provide pharmaceutical services to underserved areas and to address the problem of pharmacist shortage. This paper has reviewed the multi-faceted phenomenon of telepharmacy, summarizing different experiences in the area. Advantages and limitations of telepharmacy are discussed as well. *Materials and Methods*: A literature analysis was carried out on PubMed, using as entry term “telepharmacy” and including articles on the topic published between 2012 and 2018. *Results:* The studies reviewed were divided into three categories of pharmacy practice, namely (1) support to clinical services, (2) remote education and handling of “special pharmacies”, and (3) prescription and reconciliation of drug therapies. In general, different telepharmacy services were effective and accompanied by a satisfaction of their targets. *Conclusions*: Nowadays, the shortage of health personnel, and in particular pharmacists, is a challenging issue that the health systems have to face. The use of a new technology such as telepharmacy can represent a possible option to solve these problems. However, there are unsolved limitations (e.g., legal implications) that make greater diffusion of telepharmacy difficult. Stronger data on the effectiveness of this area of pharmacy care, together with a critical evaluation of its limits, can make actors involved aware about the potentialities of it and could contribute to a larger diffusion of telepharmacy services in the interest of communities and citizens.

## 1. Introduction

Pharmacies are important structures of the health systems and can offer health services in a capillary way due to their wide diffusion at least in industrialized countries. Qualified health professionals such as pharmacists, besides dispensing medicinal products, can give advice to patients on drug assumption regimens and can also offer pharmacovigilance services [1].

In spite of the relevant role of pharmacies as first level health care points, an uneven distribution of these structures is noticeable also in developed countries, with shortage in their distribution at a regional level and across urban and rural areas [2]. These problems can become more relevant in the near future in view of the expected decrease in the number of pharmacists [3].

Application of the information and communication technologies (ICTs) to the health sector can open new perspectives in the delivery of health services and can contribute to limit the problem of decreased availability of health professionals. One opportunity can be represented by telepharmacy services. Telepharmacy is defined as “the provision of pharmacist care by registered pharmacists and pharmacies through the use of telecommunications to patients located at a distance” [4]. Telepharmacy services already developed include medication selection, order review and dispensing, patient counselling and monitoring, and provision of clinical service [4,5]. A typical feature of a telepharmacy service is that the pharmacist is not physically present at the point of pharmacy operations or patient care. Advantages of telepharmacy services are represented by a wide coverage of the pharmaceutical service also in areas underserved due to economic or geographic problems. A decreased human interaction between health professionals and patients, problems in the evaluation of drug dispensing, and an increased risk for security and integrity of patient data represent some potential disadvantages of telepharmacy [6,7]. Telepharmacy experiences are available in some countries such as the United States, Spain, Denmark, Egypt, France, Canada, Italy, Scotland, and Germany as pointed out in this review.

The present work has analysed the main studies reporting telepharmacy experiences with particular attention to those in some way different to the conventional pharmaceutical service. This to identify new areas in which telepharmacy could increase availability of health services. Advantages and still unsolved limitations of telepharmacy practice were also discussed.

## 2. Material and Methods

This study consisted in a literature analysis carried out between January and February 2019. The research was conducted using the Pub Med library and as entry term “telepharmacy”. Selection was limited to English language articles published between 2012 and 2018 and excluded review articles.

This search resulted in 64 papers. A further analysis was performed by two researchers independently using a conventional approach consisting in reading the title and the abstract of the retrieved papers. Moreover, articles were evaluated with standard criteria of the Newcastle–Ottawa Scale (NOS) [8]. Overall studies quality was defined as poor (score 0–4), moderate (5–6), or good (7–9). Forty-six articles were excluded from a further analysis because they were not relevant to the topic or did not reach a good score based on the above criteria. The remaining 18 papers which were in line with the above inclusion criteria were analysed in detail.

## 3. Results

The selected articles based on their contents were grouped in (1) support to clinical services, (2) remote education and handling of “special pharmacies”, and (3) prescription and reconciliation of drug therapies. The main findings reported in the different papers are summarized below. The main characteristics of papers (first author and country investigated, type of study, specific purposes and sample size, strengths and weakness) are summarized in a tabular format per each group of analysis (Table 1, Table 2 and Table 3).

### 3.1. Support to Clinical Services

#### 3.1.1. Medication Adherence

The Pharmacological Intervention in Late Life (PILL) program is a service developed for veterans living in rural areas in Maine (United States) to help them to follow prescriptions adherence after hospital discharge. Geriatric patients are treated with several medicines per day (polymedication) and this articulated pharmacological treatment may cause several iatrogenic problems. PILL was designed to make easier patient medication management by assisting them with pharmacist telephone calls. In case of problems the PILL pharmacist could contact directly the primary care team to recommend eventual inappropriate interactions of treatments or potentially inappropriate therapies [9].

#### 3.1.2. Clinical Pharmacist Shortage

Clinical pharmacists are healthcare practitioners trained to ensure medication-related assistance to hospital personnel and patients. The access to this service in rural and/or remote areas is a challenge for health systems [10]. The Mount Isa Hospital [10] and the Nebraska Medical Centre [11] have deployed a remote pharmacist intervention to support underserved hospitals with proper pharmacist assistance for safe treatments to hospital inpatients.

#### 3.1.3. Pharmaceutical Counselling Activity

Home drug delivery (HDD) is a recently developed way of medicines delivery consisting in dispatching medicinal products directly at home or at the workplace of patients. This allows time and money saving, especially for patients under chronic pharmacological treatment and going often to a pharmacy or a hospital to get their medicines. HDD is of great interest and utility primarily in rural or in areas with relevant geographic dispersion [12]. In Spain, this service was offered to HIV (Human Immunodeficiency Virus) patients and was managed by hospital pharmacists [12]. An in part similar initiative was developed in Denmark [13]. This consisted in provision of remote pharmacist counselling for patients who obtained drugs via Internet or received them home. This counselling was provided mainly via telephone or video calls by community pharmacists [13]. Both experiences reached the goals of guaranteeing adequate treatment of patients. Not negligible results were money and time saving [12] and patient satisfaction [13].

### 3.2. Remote Education and Handling of “Special Pharmacies”

#### 3.2.1. Medical Staff Training and Patient Education

Telepharmacy training has been established between the St. Jude Research Hospital (Memphis, TN, USA) and the Children’s Cancer Hospital in Cairo (Egypt). This centre was opened in July 2007 and the staff had to be trained. To increase the Egyptian staff’s education, a team of pharmacists of the St. Jude Research Hospital, shared their know-how on paediatric oncology primarily via videoconferencing [14].

Telepharmacy approaches were also used applied to educate patients suffering from pulmonary diseases, namely asthma [15,16] and chronic obstructive pulmonary disease (COPD) [17], to improve medications use and adherence to treatment.

#### 3.2.2. Remote Surveillance of Anti-Neoplastic Medication Preparation

Telepharmacy was used to supervise the preparation of anti-neoplastic medications. With this approach a remote pharmacist controlled the activity of technicians via a camera system during anti-neoplastic treatments preparation. This kind of approach was used in France and involved two hospitals, namely the La Rochelle Hospital Complex (La Rochelle) and the Institute Paoli Calmettes (Marseille). Cameras were placed outside the working area to avoid contamination of preparations [18]. A similar telepharmacy service was developed in the Community Cancer Network of Alberta (Canada). This study was followed by a provincial initiative of using regularly thins kind of approach as a standard in preparing oncological treatments [19].

#### 3.2.3. Control of Medicine Chests in Seagoing Vessels

Merchant (cargo) ships do not carry on board qualified health personnel. Medical duties (including ship pharmacy maintenance) belong to the responsibility of the ship’s captain or of another officer he has delegated. Supervision and maintenance of the ship pharmacy (named ship’s medicine chest) is difficult due to the limited pharmacological/pharmaceutical skills of ship officers. The PARSI software, developed by the International Radio Medical Centre (Centro Internazionale Radio-Medico, C.I.R.M.) in Rome, makes easier to check and control the proper care of the ship pharmacy by a pharmacist ashore [20]. The software includes two sections: medicines and medical devices. The software registers medicines and medical devices withdrawn and sends a warning if replacement is needed. A feature of PARSI is that it does not require an internet connection to work. This is a practical advantage, considering that ships cannot have stable internet connections everywhere [20].

### 3.3. Prescription and Reconciliation of Drug Therapies

Remote prescription checking. In 2010, the Catholic Health Initiatives (CHI) in partnership with the North Dakota Telepharmacy Project (NDTP) started a project designed to create a Central Order Entry (COE) site in Fargo. The purpose of this service was to check prescriptions in medically underserved rural communities. The study involved 17 critical access hospitals (CAH), located in rural areas of North Dakota (*n* = 10) and North Western Minnesota (*n* = 7). The COE worked as a hub where pharmacists reviewed prescriptions, oversaw medication preparations, performed remote order entry. Activity was followed if necessary by teleconsultations with nurses, physicians, pharmacy technicians and patients [21].

In California (USA), an analysis has assessed benefits of remote review of medication orders in three small community hospitals lacking a 24-h pharmacy service. The service provided a review of medication orders before dispensing drugs from an automated cabinet and to prevent eventual medication-related problems. The nurses in charge of treatment administration could contact a pharmacist if they had questions about drugs [22].

Medication reconciliation checking. Medication reconciliation is the process of comparing the medications that a patient is taking (or should take) with newly prescribed medicinal products. This to solve discrepancies and/or to avoid possible unfavourable interactions” [23]. The medication reconciliation checking represents another area of pharmacist activity. It could be done remotely and can contribute significantly to avoid unwanted drug interactions and their consequences. The staff of the Sibley Memorial Hospital–John Hopkins Medicine, a community hospital in Washington (D.C., USA), has realized that providing to patients partially handwritten and not enough verified discharge medication list cannot guarantee safe pharmacotherapy. They have therefore requested the introduction of a telepharmacy service for medication reconciliation. With the pharmacy reconciliation program, the telepharmacists were able to support the onsite clinical pharmacists and to ensure coverage during evenings, weekends and holidays [23].

Drug dispensing supervision by pharmacy staff. Dispensing activity is one of the most common pharmacist’s tasks in community pharmacies. Mistakes should be avoided because of the potentially harm effects for patient health. The unavailability of a 24-h pharmacy service means that patients do not have the opportunity of receiving a continuous prescription check service if need arises. In this context, telepharmacy can help.

In North Dakota, a study has demonstrated that videoconferencing supervision by a remote pharmacist is effective for avoiding and/or preventing mistakes in structures staffed only by technicians. Hence, telepharmacy could represent a relevant approach to deliver and ensure a proper pharmacy service in remote sites [24].

A Telepharmacy Robotic Supply Service (TPRSS) was implemented in a rural area in North-east Scotland, to supervise drug dispensation by using a videoconferencing system [25]. The technology used was similar to that applied in North Dakota. In this Scottish experience a direct interaction between the patient and the remote pharmacist was possible via a video call system. This approach was necessary as in the site a community pharmacy was not available. Both patients and pharmacy staff evaluated positively this service, in spite of some barriers mainly due to implementation costs and increased workload. In general, the study demonstrated the usefulness of this technology in overcoming healthcare inequalities in a rural setting [25].

Another positive drug remote supervision was experienced in Germany. A Tele-intensive care unit was implemented to provide telepharmaceutical expert consultation in remote areas to identify drug related problems. The project demonstrated the efficacy of this system in improving patient outcomes and medication safety [26].

## 4. Discussion

Patients who live in rural areas or in areas of difficult access for any reasons may have difficulties to use pharmacy services [27]. For this people advances in technology may allow to reduce inequalities in healthcare delivery [28]. Countries with the largest experience in reducing the shortage in health service for these citizens are the United States and Australia [25]. The first, although rudimental, attempt to integrate a form of telepharmacy in the Australian health system is represented by the Australia’s Royal Flying Doctor Service in 1942 [29]. A more articulated experience in a telepharmacy service began in the early 2000s in North Dakota (USA) as a response to the closure of a great number of rural pharmacies [5]. After these experiences, telepharmacy is becoming to be a reality in the scenario of delivery of pharmacy services and some companies are developing and marketing assistance services which include telepharmacy provision. An example of this approach is represented by the PipelineRx and Comprehensive Pharmacy Service [30]. The interest in this new approach to pharmaceutical care is increasing, although there are still several unsolved problems that must be taken into account.

Telepharmacy diffusion is hampered by regulatory differences between countries or, as in the case of the United States, between states [31]. The first attempt to regulate the telepharmacy in the United States dates back to 2001 when the Board of Pharmacy of North Dakota drafted a code of rules to create a regulatory framework in support to the pilot study North Dakota Telepharmacy Project [32]. This first experience was followed by other laws trying to regulate the increasing possibilities offered by technological progress [5]. Today in the United States, 23 states have a legislation dedicated to telepharmacy, six states are testing pilot-studies in the perspective of telepharmacy adoption, and five states are planning to modify or have already modified existing rules for accessing to telepharmacy services [33]. The remaining 16 states have not yet considered legislative regulations for this area [33]. In terms of the regulatory framework, it should be pointed out that rules issued by different states are from time to time inconsistent and there are differences in the procedures required to obtain by local competent boards the approval for delivering a telepharmacy service [32]. Discrepancies include geographic and facility restriction, educational programs, permitted providers, staffing requirements and interstate accessibility [5,33]. To contribute to reduce uncertainty, the American Society of Health-System Pharmacists (ASHP) has stimulated a standardization process in the United States [34] and the National Association of Boards of Pharmacy (NABP) has drafted standard guidelines for telepharmacy services to facilitate the diffusion of these services throughout the United States [5].

European institutions did, to some extent, make efforts in regulating the different aspects of telemedicine, but there is still a lack of uniformity of the rules between the member states, which have kept at the national level these competences [35,36]. For instance, in Italy in 2010, pharmacies were allowed to perform clinical tests and to share patient health data with physicians, using certified networks [37]. Moreover, in 2012 the Italian Ministry of Health published their guidelines for the application of telemedicine in Italy [38]. No similar initiatives are so far available for other member states.

Increasing evidence suggests possible benefits that telepharmacy and implementation of this technology can provide to the access to healthcare services in remote communities [39]. The development of telepharmacy services is no without costs for the health systems [39], but, it is cheaper than to open new pharmacies [40]. The introduction of telepharmacy can also bring to a reduction of pharmacy services costs as with this technology one pharmacist can cover multiple sites and a wider area that otherwise would require a higher number of pharmacies [39,40]. Telepharmacy allows patients to save money and travel time, which are the major problems encountered, especially by old people, to reach healthcare structures [40,41].

The success of telepharmacy services depends to a greater extent to the level of technological infrastructures, such as efficient internet connections [40]. The lack of good technological standards can hamper a proper service delivery [39]. In any technology dealing with handling of health data, the protection and encryption of this information are of paramount importance to avoid data leaking [5,39].

Even if telepharmacy in perspective can reduce costs for health systems, acquisition of software and devices may be a financial burden that small rural healthcare centres could not afford [42]. Among the financial issues, the integration of telepharmacy in reimbursement programs of health systems should be considered [40]. In fact, telepharmacy services are not yet reimbursed and therefore patients have to pay for these services [39]. Another limiting factor is represented by some degree of scepticism of both health practitioners [39] and patients [40]. In some cases, a conflicting relationship between telepharmacy adoption and hospital staff was reported. For instance, a satisfaction survey performed at Community Cancer Network of Alberta (Canada) showed that the medical staff preferred to have the pharmacist onsite as if the service would have been made via telepharmacy; additional time is required for anti-neoplastic preparation and patient registration and information compared with conventional standard services. The telepharmacy service was preferred if it could reduce delays in treatment [19].

Another problem that does not help in a larger diffusion of telepharmacy is the not high quality of studies published on the topic. The limited number of controlled and/or randomized studies assessing the impact of telepharmacy on patient health, versus a high number of studies describing the procedures used was criticized [43]. This view was confirmed also by other studies [39].

In summary, the studies presented in this paper collectively give a positive evaluation of telepharmacy services and encourage their development primarily to counter pharmacist shortage. On the other hand, telepharmacy may have many applications with a high ethic value as they are focused on the support of patients that, otherwise, would not receive adequate pharmaceutical care. When asked about the level of satisfaction, patients largely appreciated benefits obtained [13,16,17].

Telepharmacy systems proved their effectiveness also in those operations that require a high level of precision such as the anti-neoplastic preparation [18]. Other examples reviewed here cover all aspects of pharmacy activity including prescription analysis, dispensation and administration of drugs and recognition of preventable errors. Medication errors contribute to 250,000 nonfatal injuries and 7000 deaths every year in the United States, and the annual cost of preventable adverse drug events averages approximately 2 billion US dollars [44]. In many hospitals, the implementation of telepharmacy has led to a decrease of medication error rates [45]. Data collected by another study showed an increased pharmacist intervention and an estimated cost saving due to the prevention of medication-related problems was $783,328 per year for three hospitals [22]. A frequent problem that can occur during a pharmacological treatment is the patient’s noncompliance which can lead to adverse effects due, for instance, to a sudden drug suspension. Non-adherence puts an additional expenditure burden for rehospitalization of US $100 to US $290 billion a year in United States, €1.25 billion in Europe, and approximately AUS $7 billion in Australia. Moreover, 10% of hospitalizations in older adults are attributable to noncompliance to prescription [46]. The PILL program demonstrated to counter this issue, decreasing in acute care utilization at 30 days post discharge [9].

Nowadays, people require a larger access to medicines because of increased share of aged population and co-morbidities [47]. This demographical change, which has involved especially high-income countries, has led to a higher demand of health workers, including the pharmacists, to meet these new needs [48]. The World Health Organization (WHO) indicated in its Global Pharmacy Workforce Reports a lower number of pharmacists worldwide, and that this trend augmented in countries with lower economic indicators [3]. European institutions have estimated a shortage of 1 million health practitioners by 2020, and that nearly 10% of them will be pharmacists [49]. This discouraging prevision will be the reality of a very near future and, indeed, this process has already started. Therefore, it is essential to deploy effective solutions rapidly. The International Pharmaceutical Federation (FIP) has acknowledged the increased use of technology as one of the key factors to tackle pharmacists’ scarcity [50]. The possibilities offered by telepharmacy are large and can represent a suitable solution to substitute an on-site pharmacist.

## 5. Conclusions

The adoption of telepharmacy can represent a solution to the problem of pharmacist shortage and can contribute to guarantee a proper pharmaceutical assistance in underserved areas. The diffusion and adoption of telepharmacy represent a challenge involving different actors; the cooperation between public and private sectors as well as scientific institutions and academia is of paramount importance to obtain relevant results and an effective improvement in healthcare.

## Figures and Tables

**Table 1:** Details on papers in support to clinical services reviewed.

First Author, Year, Country, [Reference]	Type of the Study	Specific Purposes	Sample Size	Strength	Weakness
Rebello 2017, USA, [9]	Original research	Therapy adherence after patient’s discharge.	100 veterans.	Detection of errors in over 70% of patients and decreased acute care utilization. Simplicity of application.	Not identified.
McFarland 2017, Australia, Queensland, [10]	Original research	Use of telepharmacy to deliver a clinical pharmacy service.	48 patients.	The service helped to increase the access to clinical pharmacy service.	The service efficiency is affected by the hospital staff presence.
			55 medication action plans.		
					Technical difficulties.
					Two sessions per week were not sufficient to carry out a proper patient follow-up.
			106 inpatient reviews.		
Sankaranarayanan 2014, USA, [11]	Retrospective study	Assistance delivered by pharmacists of Nebraska Medical Centre to several rural hospitals.	450,000 prescriptions.	Appreciation of this telepharmacy service demonstrated by an increased number of interventions from the first year onwards.	Workload increase for pharmacists because of a higher number of consultations required.
Margusino-Framinian 2019, Spain, [12]	Original article	Pharmacist teleconsultation.	38 patients	Treatment goal achieved.	HIV (Human Immunodeficiency Virus) patients included in the study had already shown to be adherent to treatments.
				Money saving.	
				High level of satisfaction among patients.	
Ho 2015, Denmark, [13]	Original research	Telepharmacy counselling service.	500 consecutive chat transcripts	High level of satisfaction among patients who answered the two optional questions about satisfaction.	Not identified.

**Table 2:** Specific telepharmacy experiences of remote education and handling of unconventional pharmacies.

First Author, Year, Country, [Reference]	Type of the Study	Specific Purposes	Sample Size	Strength	Weakness
Alfaar 2012, Egypt–USA, [14]	Original research	Application of telepharmacy for staff training.	106 surveys.	General satisfaction among attendees.	Interference with Internet functions.
					A change of IP (Internet Protocol) and gateway caused issues.
					Background noise due to microphone settings.
Brown 2017, USA, [15]	Clinical report	Patient education on asthma treatment.	20 patients.	This service was useful to help patients to control their therapy.	Technologies used.
					Appropriate location.
					Pharmacists must be properly trained.
Young 2012, USA, [16]	Pilot study	Assistance performed by the pharmacist to help therapy adherence for asthma patients	98 patients.	Almost all patients who completed the survey about satisfaction gave a positive opinion.	Not identified.
Margolis 2013, USA, [17]	Letter to the editor	Education for patients in the use of inhaled medications for chronic obstructive pulmonary disease (COPD)	97 patients.	General satisfaction and improved inhaler use.	Not identified.
Benizri 2016, France, [18]	Original research	Check of prescription and preparation of chemotherapy medications.	120 vials.	Elevated level of sensitivity, specificity and accuracy. Low graduation errors.	Operators require a training period to learn the correct process and not to skip any step during preparation. Syringe graduation is difficult to read in case of coloured and dark drugs.
Gordon 2012, Canada, [19]	Original research	Remote check of chemotherapeutic preparations.	47 patients; 109 treatment visits;	The 100% of patients, nurses, physicians and pharmacy staff preferred telepharmacy instead of treatment delay.	Need for extended time to drugs preparation and registration of patients’ data.
			248 chemotherapy preparations compounded.		
Nittari 2016, Italy, [20]	Original research	Handling of ship’s pharmacy.	Not specified.	It does not require an internet connection to work.	Not identified.

**Table 3:** Prescription and reconciliation of drug therapies.

First Author, Year, Country, [Reference]	Type of the Study	Specific Purposes	Sample Size	Strength	Weakness
Scott 2014, USA, [21]	Clinical report	Prescriptions check in hospital	17 CAHs (critical access hospitals).	Increased pharmacist’s intervention, especially to solve transcribing and prescribing errors.	Not identified.
Schneider 2013, USA, [22]	Clinical Report	Check of inpatients’ prescriptions	3888 medications.	Increased pharmacist intervention; potential adverse drug event detected; money saving.	Not identified.
Keeys 2014, USA, [23]	Case study	Check of medication reconciliation before hospital discharge.	6402 reconciled discharge medication lists.	Detection of medication discrepancies. Most of the mistakes were in unreconciled medication orders, order clarification and duplicate orders.	Increase of amount of time required for reconciliation process.
Scott 2012, USA, [24]	Original research	Detection of errors during drug dispensation using telepharmacy.	47,078 prescriptions.	Errors were more likely to be detected when the drug selling was checked by a telepharmacist.	Not identified.
Inch 2017, Scotland, [25]	Research paper	A Telepharmacy Robotic Supply Service to provide community pharmacy service to a rural area in Scotland, lacking a registered community pharmacy.	Baseline questionnaire: 156 participants.	The study has demonstrated that the permanent implementation of this kiosk is feasible and could represent a possible solution to overcome healthcare inequalities in underserved remote areas.	Data collected on the effectiveness of this service should be confirmed by a larger study.
					A longer data collection period including not only summer months would have been desirable.
			Follow-up questionnaire: 112 participants.		A more remote site to estimate the real effectiveness of the service would have been preferable.
			Follow-up resident interview: 14 participants.		
					Most of the local users were old and could have been unfamiliar with new technologies.
					Complete privacy was not achieved.
Amkreutz 2018, Germany, [26]	Original article	Telepharmaceutical expert consultation.	103 patients	The service had a positive outcome and improved the medication safety.	Small sample size.
					Only two drug related problems (DRPs) considered.
					Only one pharmacist involved in the identification of DRPs.
